# DDT and Malaria Prevention: van den Berg Responds

**DOI:** 10.1289/ehp.0901276R

**Published:** 2010-01

**Authors:** Henk van den Berg

**Affiliations:** Laboratory of Entomology, Wageningen University, Wageningen, the Netherlands, E-mail: henk.vandenberg@wur.nl

In their letter, Tren and Roberts raise a number of issues. The stakes in the use of DDT (dichlorodiphenyltrichloroethane) are high in terms of both malaria control and the side effects on human health and the global environment. In my review (van den Berg 2009), I attempted to balance malaria-control objectives and the risks of side effects. The manuscript was extensively reviewed by environmental and health experts even before being submitted to *EHP*. Therefore, the review is neither a case for or against the use of DDT.

The benefit of DDT in protecting people against malaria infection is beyond doubt. Therefore, any decision to replace DDT with alternatives must be based on evidence of the risks and benefits. The more we learn about DDT and its alternatives, the more critical we have to become in decision making.

Regarding health effects of DDT, Tren and Roberts point out correctly that clear and unambiguous cause–effect relationships have been lacking. However, this should not be interpreted as a lack of risk. Studies have depended mostly on epidemiologic data, many using case–control studies but lacking a solid control group. A major difficulty has been to establish differences in the level and period of past exposure, a prerequisite for hypothesis testing. For example, despite many previous studies, only recently has breast cancer been attributed to past DDT exposure (Cohn et al. 2007), but some caution with interpretation is still warranted. In addition, in a contemporary review of 494 studies, Eskenazi et al. (2009) concluded that there is a growing body of evidence that exposure to DDT and DDE (dichlorodiphenyldichloroethylene) may be associated with breast cancer, diabetes, decreased semen quality, spontaneous abortion, and impaired neurodevelopment in children.

Exposure to DDT in relation to indoor residual spraying (IRS) is of particular concern. In my review (van den Berg 2009), I referred to a recent study from South Africa (Aneck-Hahn et al. 2007) that showed a very high body burden of DDT in men living in houses routinely sprayed with DDT. Tren and Roberts highlight the potential of DDT to accumulate in the domestic environment, the location where human contact with DDT is likely to occur. Notably, data on exposure and health effects in young children, pregnant women, and other susceptible groups are still lacking in relation to IRS. At the time of my review, the only available data on health effects were on semen quality (Aneck-Hahn et al. 2007), which I used merely as an indication of health effects in relation to DDT use in IRS. I did not speculate on the impact of semen quality on human fertility or population growth.

Regarding environmental effects of indoor residual spraying with DDT, I quoted recent studies that reported on releases of DDT into the environment, not just in the domestic environment. Nevertheless, I pointed out that these studies need verification. Any alternatives to DDT need to be subjected to an evaluation of the side effects, especially when they involve drastic measures such as drainage of wetlands. Most alternative methods, however, have minor environmental effects (Rozendaal 1997).

In response to comments of Tren and Roberts on insecticide resistance, I need to verify two points. First, keeping vector populations under control by reducing proliferation may prevent or delay the onset of resistance development in the adult stage, but this requires further study. Second, in my review (van den Berg 2009), I mentioned that a repellent effect of DDT will reduce the risk of resistance development.

Tren and Roberts question whether decentralization can benefit malaria vector control. Indeed, the logistic requirements of IRS make this intervention particularly suitable for vertical programs, and as I pointed out in my review, it will be a major challenge to conduct and sustain IRS in a decentralized setting. Still, the experience from South Africa shows that a central program of vector control can coexist with a decentralized health system (Biscoe et al. 2005). Moreover, in Zambia, spray operators are drawn from local communities (Chanda et al. 2008). The key is to harness the potential of decentralization for vector control while providing support for IRS, where necessary. In the context of integrated vector management (IVM), the process of systems analysis, decision making, and monitoring favors a setting that is decentralized, allowing the development of a locally tailored vector control strategy and involving local actors. Barat (2006) provided a useful analysis of the success of four decentralized programs, even though, as pointed out by Tren and Roberts, the actual benefits in terms of a reduction in malaria cases may have been overstated.

In their final comment, Tren and Roberts dismiss the contribution of environmental management and other nonchemical methods in a malaria elimination strategy. When transmission reaches moderate to low levels, the main interventions will gradually be targeted only to high-risk areas, causing a reduction in the use of chemical insecticides. At decreasing transmission levels, alternative methods that reduce vector populations (e.g. environmental management, larval control) will increase in relative importance. At low levels of transmission, the human population will lose its immunity to malaria; consequently, a decrease in vector density is expected to cause a decline in malarial disease. As I indicated in my review (van den Berg 2009), modeling studies have predicted an important incremental effect of alternative methods when used in conjunction with ITN or IRS, even under conditions of intense transmission.
